# Establishment of Proliferative Tetraploid Cells from Normal Human Fibroblasts

**DOI:** 10.3389/fonc.2013.00198

**Published:** 2013-08-01

**Authors:** Susumu Ohshima, Atsushi Seyama

**Affiliations:** ^1^Division of Morphological Science, Biomedical Research Center, Saitama Medical University, Iruma, Saitama, Japan; ^2^Department of Pathology, International Medical Center, Saitama Medical University, Iruma, Saitama, Japan

**Keywords:** polyploidy, chromosomal instability, human fibroblasts, mitotic shake-off, demecolcine

## Abstract

The chromosomal instability of polyploid cells, which leads to the formation of aneuploid cells, is causally related to carcinogenesis in human tissues. However, the precise link between the chromosomal instability of polyploid cells and oncogenic transformation of them remains elusive. This is partly because we lack an experimental model in which non-transformed polyploid human cells can propagate *in vitro*. In a previous report, we demonstrated that proliferative tetraploid cells can be established from TIG-1 human fibroblasts by treatment with the spindle poison demecolcine (DC, colcemid) for 4 days. However, this procedure could not be applied to other human fibroblast strains because the resulting cells proliferated as a mixture of diploid and tetraploid populations. Here, we report a modified procedure to establish proliferative tetraploid cells from human fibroblasts of the BJ strain with minimum contamination by diploid cells. In the modified procedure, DC-arrested mitotic cells were collected by mitotic shake-off and treated with DC for an additional 3 days. DC-treated cells restarted proliferation as tetraploid cells after several days of growth arrest and showed similar growth to that of untreated diploid cells. The MDM2 antagonist Nutlin-3a activated p53 in established tetraploid cells and suppressed their growth, indicating that these cells have functional p53. These results contradicted the hypothesis that p53 functions as the tetraploidy checkpoint and prevents proliferation of tetraploid cells. Tetraploid cells established by our method could be a valuable model for the study of chromosomal instability and the oncogenic potential of polyploid cells.

## Introduction

In the early stages of human carcinogenesis, polyploid (mostly tetraploid) cells often arise and form preneoplastic lesions that give rise to malignant aneuploid cells. Although near-diploid aneuploidy is also prevalent in tumors and polyploidy is not the only route to generate aneuploid cells (1), recent research suggests that the formation of polyploid cells and their chromosomal instability appear to be causally related to human carcinogenesis. For example, increased tetraploid populations in Barrett’s esophagus predict progression to aneuploidy and esophageal carcinoma (2, 3). Tetraploidy and chromosomal instability are early events during cervical carcinogenesis and predispose cervical cells to aneuploidy (4, 5). However, the precise relationship between the chromosomal instability of polyploid cells and oncogenic transformation remains elusive. This is partly because we lack a suitable experimental model in which non-transformed polyploid human cells can propagate *in vitro*.

In a previous study, we demonstrated that proliferative tetraploid cells can be established from non-transformed human fibroblasts (TIG-1) by treatment with the spindle poison demecolcine (DC) (6). TIG-1 cells treated with DC for 4 days resumed proliferation at approximately 1 week after DC treatment and became almost completely tetraploid at 2 weeks after treatment. Established tetraploid cells grew at the same rate as untreated diploid cells and showed a similar life span as the original cells. However, we were unable to apply this procedure to other human fibroblast cell strains (TIG-3, IMR-90, BJ), which were somewhat resistant to polyploidy and maintained a significant diploid subpopulation after DC treatment. In the present study, we developed a modified procedure for the induction and isolation of polyploid cells and showed that the method is effective for the establishment of proliferative tetraploid cells from BJ human fibroblasts with minimum contamination by diploid cells.

## Materials and Methods

### Cell culture

BJ normal human fibroblasts, which were originally established from normal human foreskin, at 21 population doubling levels (PDLs) were obtained from American Type Culture Collection (VA, USA). Cells were grown in minimum essential medium with α modification (MEM-α; Sigma-Aldrich Co., St. Louis, MO, USA) supplemented with heat-inactivated 10% (v/v) fetal bovine serum. Cells were incubated in a 5% (v/v) CO_2_ atmosphere at 37°C and passaged every 3 or 4 days, ensuring that cells never exceeded subconfluent density. These cells grew up to 60 PDLs or more before they reach a state of senescence. For immunofluorescent studies, cells were seeded onto sterile glass slides, placed into culture dishes, and incubated for 2 days before staining.

### Induction and establishment of polyploid cells from BJ cells

Cells under eight passages (equivalent to PDL ≤40) were used for induction of polyploidy. The method described in a previous report (6), which was based on the method reported by Fujikawa-Yamamoto et al. (7), was modified to induce the formation of polyploid cells from BJ fibroblasts. Exponentially growing BJ cells in T75 flasks were treated with medium containing 0.1 μg/ml DC. After 16–18 h of DC treatment, DC-arrested mitotic cells were collected by gentle shaking of the flask and washing with medium followed by centrifugation. The time point for collection of mitotic cells was determined by preliminary observation using time-lapse recording to obtain as many mitotic cells as possible. In additional preliminary experiments, collection of mitotic cells at different time points between 0 and 24 h of DC treatment yielded the highest number of cells at 16–18 h. Therefore, this time point was used for recovery of mitotic cells in subsequent experiments. Mitotic cells collected by the shake-off method were reseeded into 60 mm culture dishes and treated with DC for an additional 3 days. Usually, cells collected from six T75 flasks were seeded into one or two 60 mm culture dishes, subjected to additional DC treatment, and then incubated in drug-free medium. Cells were harvested at the indicated time points, and DNA content was analyzed with a BD CycleTEST™ Plus DNA Reagent Kit (BD Biosciences, USA) and a BD FACSCanto II flow cytometer (Becton Dickinson, USA).

### Chromosome analysis and fluorescence *in situ* hybridization

Chromosome slides were prepared by standard procedures after cells were treated with DC for 6 h. At least 100 metaphase cells per time point were digitally photographed, and the chromosome number per cell was scored using image analysis software (ImageJ 1.44p) after manual separation of touching and overlapping chromosomes using the photo editing software Photoshop Elements. For fluorescence *in situ* hybridization (FISH) analysis, cells were processed and fixed as for chromosome analysis. Fixed cells were heat-denatured and hybridized with a SpectrumOrange-labeled centromere probe for chromosome 11 and a SpectrumGreen-labeled centromere probe for chromosome X (Abbott Molecular Inc. Des Plaines, IL, USA) according to the manufacturer’s instructions. Signals for chromosome 11 and X in at least 1000 interphase cells per experiment were scored using a fluorescence microscope equipped with appropriate filters.

### Analysis of centrosomes

We examined the number and localization of centrosomes in mitotic tetraploid cells by immunofluorescence to confirm whether bipolar divisions contribute to propagation of tetraploid BJ cells. Cells cultured on a glass slide were fixed with 2% (v/v) formaldehyde, permeabilized with 0.25% (v/v) Triton X-100, and incubated for 1 h at room temperature with mouse monoclonal antibodies against α-tubulin (Sigma T9025, 1:800 dilution) and centrin-2 (Santa Cruz SC-27793-R, 1:800 dilution), followed by Alexa Fluor 488-conjugated goat anti-mouse IgG (Molecular Probes, 1:500 dilution) and Alexa Fluor 555-conjugated goat anti-rabbit IgG (Molecular Probes, 1:500 dilution). Cells were then treated with RNase (0.5 mg/ml) containing 5 μg/ml 4′,6-diamidino-2-phenylindole (DAPI). DNA content was measured by DAPI fluorescence using a laser scanning cytometer (LSC-2, Olympus, Japan) equipped with a violet (405 nm) laser and a blue channel filter (460–485 nm). Chromosomes, mitotic spindle, and centrosomes in mitotic cells were inspected using appropriate filter sets, and localization of chromosomes and number of centrosomes were scored in at least 50 mitotic cells per time point after DC treatment.

### Analysis of p53 function

To examine the p53 status of untreated BJ cells and established tetraploid cells, the effect of Nutlin-3a (NT) on gene expression and cell growth was analyzed. NT stabilizes p53 by blocking its interaction with the E3 ubiquitin ligase MDM2, which promotes its proteasomal degradation. Therefore, NT treatment should activate p53 downstream proteins such as p21 and suppresses the growth of cells in which p53 is functional. To examine the effect of NT on p53 and p21 expression, cells seeded on glass slides in a previous day were treated with 10 μM NT for 24 h. Cells were then fixed with 2% formaldehyde solution for 30 min, permeabilized with 0.25% (v/v) Triton X-100, and incubated with mouse monoclonal antibody against p53 (clone DO-1, 1:100 dilution, Santa Cruz Biotechnology) or p21 (clone F-5, 1:200 dilution, Santa Cruz Biotechnology), followed by Alexa Fluor 488-conjugated goat anti-mouse IgG (Molecular Probes, 1:500 dilution). Cells were then treated with RNase (0.5 mg/ml) containing 5 μg/ml propidium iodide (PI) and analyzed with a LSC-2 laser scanning cytometer (Olympus, Japan). The frequency of p53 positive cells was estimated by comparing the fluorescence intensity with that of cells incubated with isotype control antibody. The effect of NT on cell growth was examined by treating cells with 10 μM NT continuously for 3 days, and cell growth was analyzed by counting cell numbers every day during the treatment.

## Results

### Establishment of polyploid cells from BJ cells

**Figure 1:**
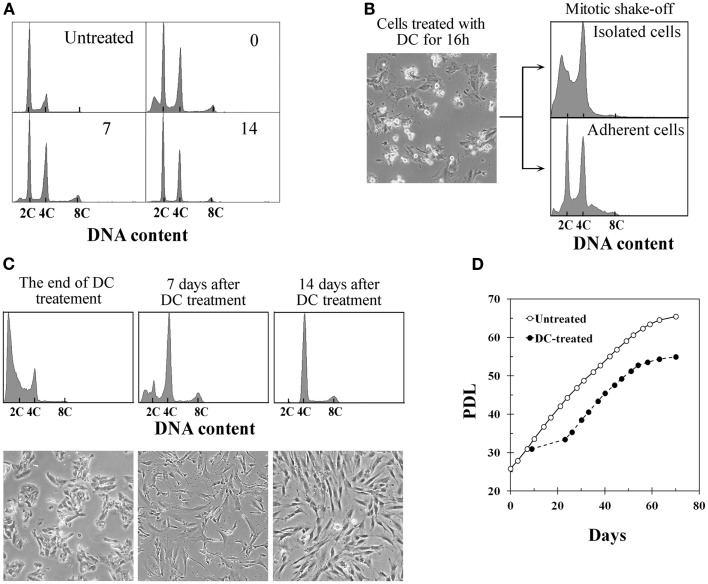
**Establishment of tetraploid cells from human fibroblasts by treatment with demecolcine**. **(A)** Changes in the DNA histograms of BJ cells treated with demecolcine (DC) continually for 4 days. Numerals in the histograms represent the time (days) after drug removal. The abscissa shows DNA content (C, complement). **(B)** Phase-contrast micrograph of BJ cells treated with DC for 16 h (left panel), and DNA histograms of mitotic cells isolated by the shake-off method (right upper panel) and adherent cells (right lower panel). **(C)** Changes in DNA histograms and morphology of cells isolated by the shake-off method. Upper panels show DNA histograms and lower panels show phase-contrast micrographs. The abscissa represents DNA content (C, complement). **(D)** Growth profiles of untreated BJ cells (open markers) and DC-treated cells (closed markers). The growth curve of DC-treated cells was made based on the observation that 10% of mitotic cells collected by shake-off had adhered to the dish at the end of DC treatment.

Treatment of BJ cells with 0.1 μg/ml DC continually for 4 days following the procedure described previously to establish tetraploid cells from TIG-1 cells resulted in a mixture of diploid and tetraploid populations (Figure 1A). On the other hand, DC-arrested mitotic BJ cells collected by the shake-off method after 16–18 h of DC treatment consisted of cells with a 4C DNA content and presumably apoptotic cells with a DNA content<2C, whereas adherent cells had 2C and 4C DNA contents as determined by DNA histograms (Figure 1B). The collected cells with 4C DNA were then treated with DC for an additional 3 days to establish polyploid cells. Additional DC treatment of less than 3 days was not sufficient to establish polyploid cells, and cells reverted to diploid status after DC treatment (data not shown). DC treatment resulted in a significant number of floating cells, and approximately 10% of the cells collected by shake-off had adhered to the culture dishes at the end of treatment. Analysis of the DNA content by flow cytometry at this time point showed 4C DNA and apoptotic cells (Figure 1C, leftmost panels). After DC treatment, cells showed growth arrest and large, flattened morphology for several days. After 5–7 days of DC treatment, small proliferating cells appeared among the large flattened cells (Figure 1C, middle panels), and cells became almost completely tetraploid at 2 weeks after DC treatment (Figure 1C, rightmost panels). Less than 5% of contaminating diploid cells was observed at this point, and these cells showed a tendency to diminish with serial passaging. The mean frequency of diploid cells at 4 weeks after DC treatment was 1.1% in three experiments. Established tetraploid cells grew at almost the same rate as diploid cells and showed an approximately equal replicative lifespan as the original diploid cells (Figure 1D). The average doubling time at 2–6 weeks after DC treatment was 44.5 ± 3.4 h in three experiments, while the doubling time of untreated diploid cells in the same period was 46.5 ± 2.6 h.

### Number of chromosomes and FISH signals after establishment of tetraploid cells

**Figure 2:**
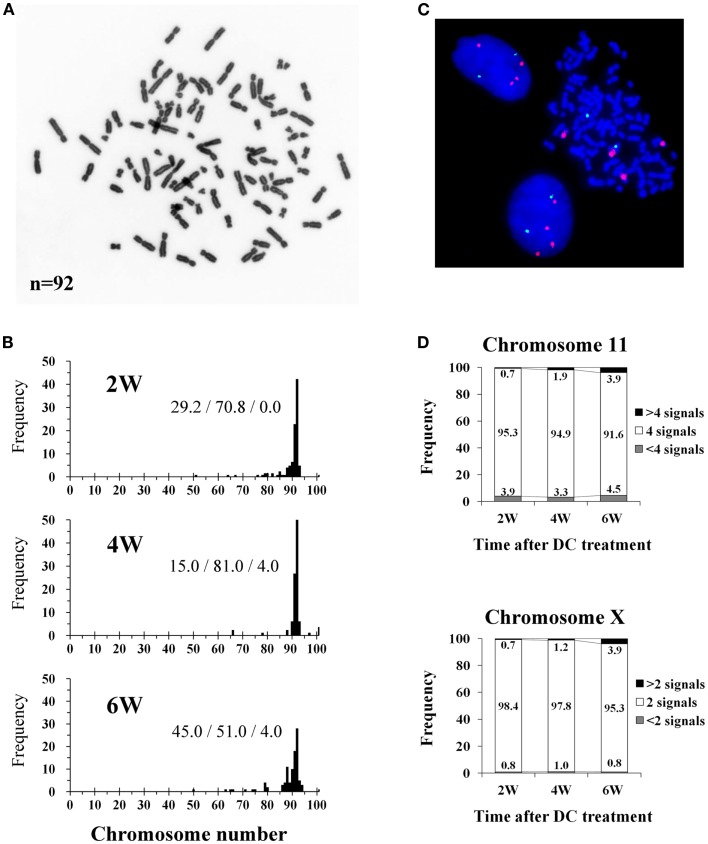
**Chromosome and fluorescence *in situ* hybridization (FISH) analysis of tetraploid BJ cells**. **(A)** Photomicrograph of chromosomes from an established tetraploid cell prepared after 16 days of DC treatment. **(B)** Histograms of chromosome number in established tetraploid cells scored at 2, 4, and 6 weeks after DC treatment. Chromosomes were scored for at least 100 mitotic cells from two experiments per time point. The numbers on the histogram bars represent frequencies of cells with<91, 91-93,>93 chromosomes respectively. **(C)** Photomicrograph of FISH in established tetraploid cells prepared at 16 days after DC treatment. Orange signals represent chromosome 11, and green signals represent chromosome X. Two interphase cells and one mitotic cell show tetrasomy for chromosome 11 and disomy for chromosome X, indicating that they are male tetraploid cells. **(D)** Frequencies of FISH signals for chromosome 11 and X scored at 2, 4, and 6 weeks after DC treatment. Signals were scored for at least 1000 interphase cells per experiment. Numbers in the accumulated bar graphs represent frequencies of cells with the given number of signals.

The mode of chromosome counts in established tetraploid cells was always 92 throughout the experimental period, and more than 80% of the cells had tetraploid chromosome counts (91–93 chromosomes) after 4 weeks of DC treatment (Figures 2A,B). However, the frequency of cells with tetraploid chromosome counts decreased to 51.0% and cells with reduced chromosome counts (<91 chromosomes) increased to 45.0% at 6 weeks after DC treatment. FISH analysis showed that approximately 95% of cells had four signals for chromosome 11, and approximately 98% of cells had two signals for chromosome X at 2 and 4 weeks after DC treatment, whereas the frequency of these cells decreased slightly at 6 weeks after DC treatment (Figures 2C,D). At 6 weeks after DC treatment, cells with<4 and>4 signals for chromosome 11 slightly increased, whereas for chromosome X, cells with>2 signals slightly increased but cells with<2 signals did not increase.

### Centrosome number and localization in tetraploid BJ cells

**Figure 3:**
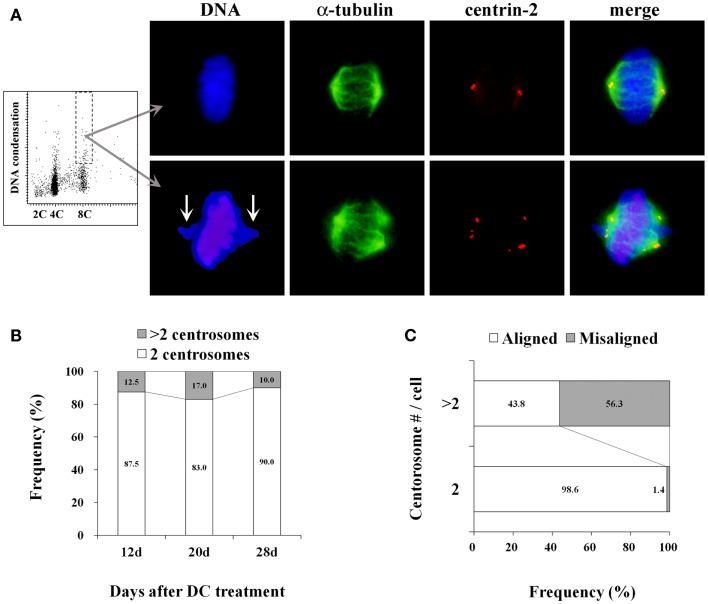
**Centrosome analysis in tetraploid BJ cells**. **(A)** A representative dot plot showing DNA content vs. DNA condensation obtained by laser scanning cytometry (leftmost panel), photomicrographs of immunohistochemically stained mitotic cells in a 8C DNA subpopulation (second to rightmost panels). Chromosomes are labeled with DAPI (*blue*), mitotic spindles are labeled with anti-α-tubulin antibody (*green*), centrosomes are labeled with anti-centrin-2 antibody (*red*). Upper panels show a metaphase cell with normal morphology, and lower panels show a metaphase cell with misaligned chromosomes (arrows), an irregular-shaped spindle and excess centrosomes. **(B)** Frequencies of mitotic cells with 2 centrosomes and excess centrosomes in established tetraploid cells. Number of centrosomes was scored in at least 50 mitotic cells per time point after DC treatment. Numbers in the accumulated bar graphs represent frequencies of cells with the given number of centrosomes. **(C)** The correlation between centrosome number and chromosome misalignment in tetraploid metaphase cells. The frequency of metaphase cells with aligned and misaligned chromosomes in pooled data from all time points shown in **(B)** was calculated according to the centrosome number. Numbers in the accumulated bar graphs represent frequencies of metaphase cells with aligned and misaligned chromosomes.

More than 80% of mitotic cells had 2 centrosomes, while 10–17% of mitotic cells had excess centrosomes in established tetraploid cells at analyzed time point after DC treatment (Figures 3A,B). This frequency of mitotic cells with excess centrosomes in tetraploid BJ cells was almost the same as the diploid BJ cells. Centrosomes in tetraploid BJ cells were usually recognized by anti-centrin-2 antibody as pairs of two dots presumably representing centrioles, although some centrosomes had more than two centrioles. While most metaphase cells with 2 centrosomes had a bipolar spindle and aligned chromosomes, metaphase cells with excess centrosomes were often accompanied by an irregular spindle and misaligned chromosomes (Figure 3A). Presence of misaligned chromosomes in metaphase cells was strongly correlated with the presence of excess centrosomes (Figure 3C).

### Activation of p53 and growth suppression by NT treatment

**Figure 4:**
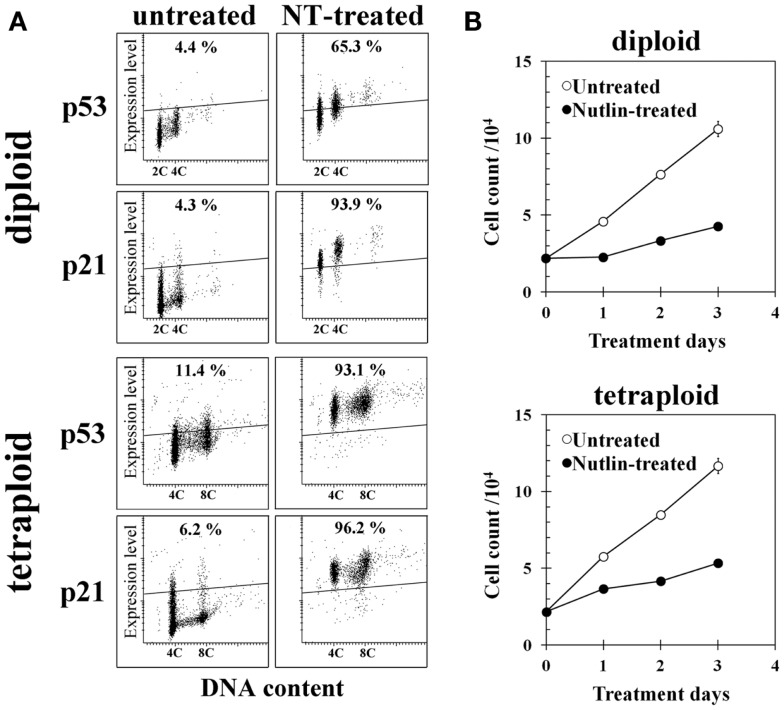
**Effect of Nutlin-3a (NT) on gene expression and cell growth of diploid and tetraploid BJ cells**. **(A)** p53 and p21 expression in untreated and NT-treated cells. Cells on glass slides were treated with 10 μM NT for 24 h and immunohistochemically stained with anti-p53 or anti-p21 monoclonal antibody, then analyzed by a laser scanning cytometer as described in the Section “Materials and Methods.” The regions indicating positive expression were drawn above the fluorescence intensity of cells incubated with isotype control antibody. Percentages in the figures indicate frequencies of cells with positive expression of each gene. 2C and 4C indicate the positions of cells with 2C and 4C DNA content. **(B)** Cell growth of control and NT-treated diploid BJ cells (upper panel) and tetraploid BJ cells (lower panel). Cells were treated with 10 μM NT continuously.

Treatment of diploid BJ cells and established tetraploid cells with 10 μM NT for 24 h resulted in the accumulation of the p53 and p21 proteins in both cell types (Figure 4A). Continuous treatment with the same concentration of NT suppressed cell growth (Figure 4B), suggesting that p53 was functional in both cell types.

## Discussion

In the present study, we showed that proliferative tetraploid cells can be established from BJ human fibroblasts by a combination of mitotic shake-off and DC treatment. The mitotic shake-off method, which is used to synchronize the cell cycle of adherent cells, is based on the fact that cells round up during mitosis and can be dislodged from plastic culture dishes by agitation (8). The collection of mitotic cells by the shake-off method enabled the effective induction of tetraploid cells in BJ cells, because most of adherent diploid cells can be eliminated by this method. The difference in the response to DC treatment between BJ cells, in which tetraploidy was not completely induced by the original method, and TIG-1 cells, which became completely tetraploid under similar conditions (6), is unknown at present. One possible explanation is that a significant population of BJ cells arrest at G1 phase by DC treatment and resume proliferation after treatment, whereas TIG-1 cells do not show G1 arrest in response to the same treatment. Further investigation is necessary to verify this hypothesis.

Several studies have shown that mammalian cells with functional p53 arrest with a 4C DNA content in response to spindle inhibitors, whereas cells lacking p53 re-replicate and become polyploid (9–11). Andreassen et al. hypothesized that the tetraploid state induces a p53-dependent arrest of non-transformed mammalian cells in G1 phase termed “tetraploidy checkpoint” (12), and this hypothesis has been supported by other investigators (13, 14). However, our results contradict this hypothesis because induced tetraploid cells showed similar growth to that of untreated diploid cells, despite the fact that they had functional p53. Induction of proliferative tetraploid cells from non-transformed human cells with functional p53 was also observed in TIG-1 human fibroblasts (6). Therefore, the cell cycle arrest with 4C DNA content in response to spindle inhibitors was probably not caused by tetraploidy itself but may have been triggered by other factor(s). The basis for this assumption is our observation that cell cycle arrest with 4C DNA content after DC treatment was transient but not permanent. In our experiment, treatment of BJ cells with DC for 4 days coupled with isolation of 4C mitotic cells by the shake-off method resulted in growth arrest for approximately 5–7 days, followed by the resumption of proliferation as tetraploid cells thereafter. Studies supporting the existence of a “tetraploidy checkpoint” observed cell cycle arrest only during drug treatment but failed to analyze growth recovery after drug treatment (9–11). In fact, several studies have shown results supporting the lack of checkpoints for tetraploidy in mammalian cells (15–19). These studies suggest that drug treatments used to synchronize cells or induce tetraploidy cause cellular damage that is likely to be DNA damage, which results in G1 arrest with a 4C DNA content. Other studies have indicated that prolonged mitotic arrest induced by various mechanisms results in DNA damage (20, 21). If the growth arrest observed after DC treatment in our study was caused by DNA damage, the resumption of cell growth after a recovery period is reasonable. We are currently performing experiments to verify this mechanism.

Although the chromosome number in established tetraploid cells was relatively stable, the number of cells with a reduced chromosome count (<91 chromosomes) increased at 6 weeks after DC treatment. This reduction of chromosome number at later passages was also observed in tetraploid cells established from TIG-1 human fibroblasts (6). These observations suggest that polyploid cells lose their chromosomes during cultivation and are converted into aneuploid cells. The propagation of aneuploid cells should be confirmed, however, because it was shown that the p53 pathway plays an important role in limiting the propagation of aneuploid human cells in culture (22).

Most of established tetraploid mitotic cells had only two centrosomes with bipolar spindles like diploid cells, and this might contribute to stable propagation of tetraploid cells and stability of chromosome number. Recent studies show that supernumerary centrosomes in cancer cells and non-transformed tetraploid cells are often clustered into two spindle poles, which enables bipolar divisions (19, 23). However, most centrosomes in established tetraploid BJ cells consisted of only two centrioles, suggesting these cells were established by mechanisms different from the clustering of excess centrosomes. These observations regarding centrosomes were exactly the same as the observations for tetraploid cells established from TIG-1 fibroblasts in the previous study (6).

The method described in this study is likely to be applicable to other fibroblast strains too, because the induction of tetraploidy in the human fibroblast strain IMR-90 was also confirmed (unpublished data). In other studies, mouse tetraploid cells were used to show the promotion of tumorigenesis by tetraploidy (24, 25); however, in these studies, fluorescence-activated cell sorting (FACS) was used to isolate tetraploid cells from diploid cells. Our method has the advantage that the induction of tetraploid cells can be achieved easily without FACS. Tetraploid cells established by our method may be of value as a model for studying the chromosomal instability and oncogenic potential of polyploid cells.

## Conflict of Interest Statement

The authors declare that the research was conducted in the absence of any commercial or financial relationships that could be construed as a potential conflict of interest.
